# Evaluating the association between lifetime physical activity and oral squamous cell carcinoma: A case-control study

**DOI:** 10.1371/journal.pone.0303929

**Published:** 2024-05-20

**Authors:** Seyyed Abolfazl Tabatabaie-Zadeh, Nazanin Mahdavi, Behnaz Mahdaviani, Maryam Selk-Ghaffari

**Affiliations:** 1 School of Dentistry, Tehran University of Medical Sciences, Tehran, Iran; 2 Department of Oral and Maxillofacial Pathology, School of Dentistry, Tehran University of Medical Sciences, Tehran, Iran; 3 Sports Medicine Research Center, Neuroscience Institute, Tehran University of Medical Sciences, Tehran, Iran; University of Hong Kong, HONG KONG

## Abstract

**Purpose:**

Oral cancers are the 6th most common malignancy worldwide and oral squamous cell carcinoma, comprises over 90% of oral cancers. This study investigates the relationship between physical activity level during the lifetime and oral squamous cell carcinoma risk.

**Methods:**

100 oral squamous cell carcinoma patients and 200 healthy individuals participated in this case-control study. Physical activity level was evaluated via the Lifetime Physical Activity Questionnaire. The occupational, household, and sports domains of lifetime physical activity were determined. Case and control groups’ participants were matched in terms of sex, age, smoking, and alcohol consumption by the Frequency Matching Method. Mann-Whitney U Test was applied to compare physical activity levels between groups.

**Results:**

The Body Mass Index was higher among cases compared with controls. The average amounts of lifetime physical activity among cases and controls were approximately identical. However, only a statistically significant difference between time spent on total lifetime physical activities and the oral squamous cell carcinoma risk was discovered. Moreover, there were no statistically significant odds ratios in examining the risk associated with each domain of activities.

**Conclusions:**

The total time spent on lifetime physical activity may decrease the risk of oral squamous cell carcinoma; However, the total level and intensity of lifetime physical activity are not significantly associated with the oral squamous cell carcinoma risk. Further studies are required in this field.

## Introduction

Engaging in regular physical activity has been indicated as a protective factor against a high spectrum of non-communicable diseases including cardiovascular disease (CVD), brain stroke, diabetes mellitus type 2, hypertension, metabolic syndrome, and cognitive diseases such as dementia and Alzheimer’s disease [1,2]. Various studies have been conducted regarding the role of physical activity in the prevention, treatment, and rehabilitation of different types of cancer [3]. The protective role of physical activity against colon, breast, prostate, lung, ovarian and endometrium cancer has been identified [4]. However, uncertainties exist regarding the association between physical activity and some other cancer origins [5].

Oral cancers are the 6th most common malignancy worldwide and oral squamous cell carcinoma (OSCC) is the most prevalent head and neck cancer (90%) [6–9]. Major determined risk factors for head and neck cancer consist of Human papillomavirus (HPV), alcohol consumption, smoking, and habitual behaviors, such as tobacco and betel nut usage, as well as inadequate daily consumption of fresh fruits and vegetables [6,7,10,11]. An unhealthy lifestyle has been proposed as a modifiable risk factor for at least 20% of cancer types. Lifestyle corrections and modifications are suggested as a preventive strategy to reduce cancer risk and progression in some cancer types [12]. Some of the suggested mechanisms for physical activity protective effect against cancer are regulation of interleukin-6 (IL-6) activity, reduction in insulin growth factor-1 (IGF-1) levels, mitochondrial function enhancement in apoptosis of cancer cells, and epigenetic alterations [13].

The role of engaging in regular physical activity has been determined as a protective factor in some cancers including breast and colon cancers [4]. However, limited evidence and controversies exist regarding the role of physical activity in reducing the risk of oral cancers [4,5]. According to the high prevalence of oral cancers and their considerable burden on the health system, developing preventive strategies including modifying lifestyle by increasing physical activity level might be a solution. Lifetime physical activity is defined as the total physical activity accomplished over the course of the individual’s lifetime across three main domains including occupational activity, sports/recreation, and household activity [14]. This study aims to evaluate the association between lifetime physical activity level and the risk of oral squamous cell carcinoma.

## Materials and methods

This study was a case-control study, which was conducted on human participants in the Faculty of Dentistry of Tehran University of Medical Sciences. The study was permitted and approved by the Tehran University of Medical Sciences ethical committee (Ethics code: IR.TUMS.DENTISTRY.REC.1400.062). All participants in the study were provided with complete data about the objective of the study. Written informed consent was obtained from all individual participants included in the study. The participants were recruited from December 22, 2021, to May 22, 2022.

The participants enrolled in this study involved case and control groups among individuals referred to the Faculty of Dentistry of Tehran University of Medical Sciences, Shariati Hospital, and Cancer Institute of Imam Khomeini Hospital. The case group consisted of 100 recently diagnosed oral squamous cell carcinoma patients who had the eligibility criteria based on inclusion and exclusion criteria. The control group comprised 200 healthy individuals. The control group was examined for any oral suspicious premalignant lesions; if detected, the participant in the control group was excluded from the study.

The inclusion criteria were as follows: patients affected by oral squamous cell carcinoma diagnosed in the last 6 months according to the histopathological criteria, ability to walk, and willingness to participate in the study. The exclusion criteria were as follows: a history of bone marrow transplantation, mental disorders, oropharyngeal cancer, wearing a complete denture, using non-smoking tobacco like betel quid, and presence of diseases such as Fanconi anemia, Xeroderma pigmentosum.

Case and control groups’ participants were matched in terms of age ±2 years, sex, smoking, and alcohol consumption by the Frequency Matching Method. We recruited two controls for each case with the same matching variables. Finally, frequency-matched case and control groups were included in this study.

In the present study, smokers were those who consumed currently at least one cigarette per day for at least 1 year. Also, alcoholics were assumed those who consumed currently two drinks of alcohol per week over a 1-year minimal period [15].

Physical activity was evaluated via the Lifetime Physical Activity Questionnaire (LTPAQ) [14]. An instructed interviewer conducted the physical activity assessments face to face. The reliability and validity of this questionnaire have been proved in a previous study on the Iranian population [16].

LTPAQ recorded the physical activity level of participants during their whole lifetime. Three distinct domains of physical activity have been explored in this questionnaire [14].

1. Occupational and volunteer activities: In this domain, the activities that have been done for at least 8 hours per week for 4 months or 128 hours per year or 2.5 hours in all weeks of the year were measured. The age at the start and end of the activity, the intensity of the action, which was numbered 1 to 4, and the type of travel-related physical activity to go to work, such as walking or cycling, were asked of the participants.
2. Household activities: This domain included activities that were carried out for at least 7 hours per week for 4 months per year or 112 hours per year or 2.15 hours in all weeks of the year. The age at the beginning and end of the activity was also questioned.
3. Sports and exercise activities: In this type, activities were mentioned that were performed for more than 2 hours per week in 4 months of each year or 32 hours per year or 40 minutes in all weeks of each year. The type of sports activity that was done at least 10 times during the lifetime was also asked. The participants were asked how they travel to school and work, whether walking, skating, running or cycling and which sports classes they participated in. Furthermore, the starting and ending ages of the activity were questioned [14].

The level and intensity of the participants’ physical activity were reported as “Metabolic Equivalent” (MET)-hours per week according to the scientific guidelines of LTPAQ [14].

### Statistical analysis

SPSS version 27 software was used to analyze data. To determine the study participants’ characteristics, descriptive statistics were applied. We examined the assumption of normal distribution using Kolmogorov-Smirnov and the Shapiro-Wilk tests. As the variables could not reveal a normal distribution, nonparametric tests including chi-square for categorical variables and Mann-Whitney for continuous variables were used to find the differences among case-control subjects. A statistically significant difference was indicated if P<0.05. We categorized time spent in physical activity (PA) and PA levels including total and specific domains of activity into 2-quantile, based on their distribution among the control subjects. A median was derived from the time spent in PA and the PA level of control participants, and participants were then divided into two groups: those who spent time in PA and had a PA level above the median, and those who spent time in PA, and had a PA level below the median. Binary logistic regression using the Enter method was used to demonstrate the amounts of Crude Odds ratios (OR) and 95%- Confidence Interval (95%-CI) for OSCC. The dependent variable was whether or not participants had been diagnosed with OSCC. The independent variables included the mean time spent in PA and the level of PA as dichotomized from above or below the median level. We defined potential confounding factors as age (continuous), sex (male or female), smoking (yes/ no), alcohol consumption (yes/no), and Body Mass Index (BMI) (continuous). The unadjusted ORs for the association between potential confounding factors and OSCC risk were measured using univariate regression analysis.

The adjusted ORs for association between time spent in PA, as well as level of PA and OSCC risk were estimated by entering either the full set of potential confounding variables (model 1), or the known confounder(s) based on the univariate analysis of the association between confounding factors and OSCC (model 2) into a logistic regression.

## Results

A total of 300 participants (case = 100, control = 200) were recruited in the current study. Although the participants were matched based on age, sex, smoking, and alcohol consumption, cases had higher BMI compared with controls. The baseline characteristics of the participants are illustrated in Table 1.

**Table 1:** Demographic and matched data of the participants.

	Case; median [IQR]	Control; median [IQR]	P value
**Age**	58 [48.25–65]	56 [50–66]	NS (0.84)
**BMI**	24.97 [22.79–27.27]	23.18 [21.53–25.14]	<0.001
	**Case; N (%)**	**Control; N (%)**	**P value**
**Female**	47 (47%)	94 (47%)	NS (0.87)
**Male**	53(53%)	106 (53%)	NS (0.87)
**Smoking**	18 (18%)	36 (18%)	NS (1)
**Alcohol**	8 (8%)	16 (8%)	NS (1)

IQR: Inter Quartile Range, N: Number, NS: Non-Significant.

Concerning OSCC location among patients, the most prevalent affected area was the tongue, followed by buccal mucosa, lip, palate, and floor of the mouth (Table 2).

**Table 2:** OSCC distribution among the case group, according to the site of occurrence in terms of anatomic location (ICD-10).

ICD-10	location	Frequency
C00.9	lip (unspecified)	13 (13%)
C01	base of the tongue	27 (27%)
C02.9	Tongue (unspecified)	10 (10%)
C06.0	Buccal mucosa	25 (25%)
C04.9	floor of the mouth (unspecified)	6 (6%)
C05.9	Palate (unspecified)	7 (7%)
C06.9	Mouth (unspecified)	12 (12%)

The average amount of lifetime PA among cases and controls were similar. The median of total lifetime PA was approximately 80 MET. Hour/week. Although our results could not demonstrate any relationship between physical activity based on the time and intensity and the risk of OSCC, as can be seen from Table 3, cases spent somewhat less time participating in physical activities in each domain or total. There is, however, only a statistically significant difference in time spent on total lifetime physical activities (P<0.001).

**Table 3:** Time spent in physical activity (Hours/week) and intensity of lifetime physical activity (MET-hours/week) among cases and controls based on the LTPAQ’s results.

Domain of physical activity		Case		Control		P Value
		Median [IQR]	Mean±SD	Median [IQR]	Mean±SD	
*Occupational and volunteer PA*	**Intensity**	29.38 [0–71.79]	45.82±60.29	31.54 [0–64.80]	41.49±41.07	0.714
	**Time spent**	13.70 [0–24.73]	16.30±17.44	16.24 [0–29.42]	17.59±14.77	0.196
*Household PA*	**Intensity**	8.23 [1.46–54.93]	32.29±40.61	8.95 [1.49–62.62]	35.81±41.80	0.423
	**Time spent**	2.55 [0.45–19.09]	10.09±12.01	2.66 [0.48–22.30]	11.97±13.37	0.282
*Sports PA*	**Intensity**	0.32 [0–3.69]	2.55±3.84	1.49 [0–3.77]	2.51±3.26	0.357
	**Time spent**	0.12 [0–1.26]	0.85±1.23	0.70 [0–1.49]	0.87±1.04	0.259
*Total lifetime PA*	**Intensity**	67.87 [45.94–92.70]	80.65±60.01	74.30 [52.85–101.51]	79.81±40.33	0.178
	**Time spent**	24.65 [18.09–32.40]	27.25±15.54	30.08 [22.56–36.76]	30.43±11.79	**<0.001**

PA: Physical Activity, IQR: Inter Quartile Range, SD: Standard Deviation.

Table 4 illustrated the unadjusted associations between potential confounding factors and OSCC risk. From the table, it can be understood that BMI was the only confounder.

**Table 4:** Unadjusted ORs for the association between potential confounding factors and OSCC risk.

Potential confounding factor	OR	95%-CI	P value
***Age***	1.001	0.98–1.02	0.955
***Sex***	0.961	0.594–1.554	0.87
***BMI***	1.17	1.07–1.26	<0.001
***Smoking***	1	0.535–1.87	1
***Alcohol***	1	0.413–2.42	1

The results of correlational analysis providing us with the OSCC risk associated with each type of activity are set out in Tables 5 and 6. In model 1, the adjusted OR was 0.367 (95%-CI: 0.207–0.650) among subjects in the highest 2-quantile, above the median, (>30.08 hour/week) versus the lowest 2-quantile (<30.08 hour/week) of time spent PA. As a result of removing the factors that were known to be non-significant based on univariate analysis (model 2), the adjusted OR remained robustly significant. However, there were no statistically significant ORs in examining the risk associated with each domain of activities (P>0.05). Furthermore, we did not find a significant OR when comparing the highest to lowest 2-quantile of lifetime PA, neither in total nor by domain. Additionally, all multivariate regression analyses indicated that increasing BMI could increase the OSCC risk by 1.16 (95%-CI: 1.07–1.26), independently (P<0.001)

**Table 5:** Odds ratios for OSCC based on time spent in lifetime physical activity.

Time spent in PA (Hours/Week)	Cases (n)	Controls (n)	Crude			Multivariable adjusted*		
						Model 1^a^		Model 2^b^
			OR	95%-CI	OR	95%-CI	OR	95%-CI
***Occupational and volunteer PA***								
<16.24	55	100	0.818	0.505–1.324	0.650	0.327–1.292	0.853	0.521–1.399
>16.24	45	100						
***P value***			0.414		0.219	0.529		
***Household PA***								
<2.66	51	100	0.961	0.594–1.593	1.158	0.294–4.564	0.940	0.574–1.539
>2.66	49	100						
***P value***			0.870		0.834	0.807		
***Sports PA***								
<0.70	60	100	0.680	0.418–1.107	0.635	0.373–1.083	0.683	0.415–1.125
>0.70	40	100						
***P value***			0.121		0.095	0.135		
***Total lifetime PA***								
<30.08	71	100	**0.408**	**0.244–0.682**	**0.367**	**0.207–0.650**	**0.439**	**0.260–0.741**
>30.08	29	100						
***P value***			**<0.001**		**<0.001**	**0.002**		

N: Number, OR: Odds Ratio, PA: Physical Activity, 95%-CI: 95% Confidence Interval.

^a^ Multivariable adjusted for age, sex, smoking and alcohol statuses, and BMI.

^b^ Multivariable adjusted for BMI.

**Table 6:** Odds ratios for OSCC based on intensity of lifetime physical activity.

Lifetime PA (MET-Hour/Week)	Cases (n)	Controls (n)	Crude	Multivariable adjusted*
				Model 1^a^	Model 2^b^
			OR	95%- CI	OR	95%- CI	OR	95%- CI
***Occupational and volunteer PA***								
<31.54	52	100	0.923	0.571–1.492	0.854	0.442–1.653	0.98	0.598–1.606
>31.54	48	100						
***P value***			0.744		0.640	0.937		
***Household PA***								
<8.95	50	100	1.000	0.619–1.616	1.434	0.398–5.169	0.976	0.569–1.597
>8.95	50	100						
***P value***			1.000		0.582	0.922		
***Sports PA***								
<1.49	54	100	0.852	0.527–1.378	0.827	0.490–1.395	0.861	0.526–1.410
>1.49	46	100						
***P value***			0.514		0.477	0.552		
***Total lifetime PA***								
<74.30	56	100	0.79	0.485–1.273	0.84	0.492–1.434	0.87	0.529–1.429
>74.30	44	100						
***P value***			0.327		0.523	0.581		

N: Number, OR: Odds Ratio, PA: Physical Activity, 95%- CI: 95% Confidence Interval.

^a^ Multivariable adjusted for age, sex, smoking and alcohol statuses, and BMI.

^b^ Multivariable adjusted for BMI.

## Discussion

In this study, we investigated the relationship between the lifetime physical activity level and the risk of OSCC using LTPAQ. The results of the current study demonstrated that the total time spent on lifetime PA might lower the risk of OSCC; However, there was no remarkable relationship between the total level and intensity of lifetime PA and the risk of OSCC. Furthermore, different domains of lifetime PA including occupational, household, and sports PA showed no significant association with the risk of OSCC development.

In a study by Leitzmann et al., a prospective study was conducted on 487,732 participants and the association between physical activity and head and neck cancer was investigated. The results indicated that 1,249 participants were diagnosed with head and neck cancer in the oral cavity, larynx and pharynx at 42.0%, 32.5%, and 18.9%, respectively during follow-up. Low-level physical activity and moderate to high physical activity indicated no association with head and neck cancer risk compared with no physical activity with light activity (Relative Risk (RR) = 1.07; 95%-Confidence Interval (CI) = 0.83–1.39, RR = 0.81; 95%-CI = 0.64–1.03, respectively). Following adjustment for age, sex, and smoking RRs of cancers of the oral cavity, larynx, and pharynx, comparing the physical activity level from high to low were 0.98 (95%-CI = 0.75–1.29), 0.82 (95%-CI = 0.59–1.13), and 0.70 (95%-CI = 0.45–1.08), respectively [17]. The findings of a study by Leitzmann et al. were similar to our findings, and physical activity indicated no association with head and neck cancer.

In a study by Irmawati et al., the impact of moderate-intensity exercise on the ratio of Bax/Bcl-2 expression in mice injected with Benzopyrene was evaluated. Benzopyrene is a carcinogenic ingredient in cigarettes. Moderate-intensity swimming for twelve weeks led to an increase in the Bax/Bcl-2 ratio in oral squamous epithelial cells. It is suggested that an increase in the ratio of Bax/Bcl-2 matches the apoptosis process since higher expression of Bax compared to Bcl-2 leads to intrinsic apoptosis [18]. In another study by Irmawati et al., it was indicated that moderate exercise increases the expression of caspase-3 in mice injected with Benzopyrene. Caspase-3 activates CAD (Caspase-Activated DNase) endonuclease and via developing apoptotic bodies, the creation of the transformed epithelial cells of the oral squamous cell is prevented [19]. In conclusion, moderate-intensity exercise in mice led to an increased Caspase-3, and the Bax/Bcl-2 ratio, which induces apoptosis [20,21]. Irmawati et al. suggested that physical activity reduces the risk of oral cancer occurrence by diminishing the formation of transformed cells and decreasing the expression of the p53 mutant [21].

In a study by Moore et al. in 2016 based on 12 prospective cohorts, a total of 1.44 million individuals with self-reported physical activity were recruited. Patients with cancer were 186 932 individuals. High recreational physical activity level compared to low physical activity level reduced risks of colon cancer (Hazard Ratio (HR), 0.84; 95%- CI, 0.77–0.91), breast cancer (HR, 0.90; 95%- CI, 0.87–0.93), head and neck cancer (HR, 0.85; 95% -CI, 0.78–0.93), oesophageal adenocarcinoma (HR, 0.58; 95%- CI, 0.37–0.89), rectal cancer (HR, 0.87; 95%- CI, 0.80–0.95), lung cancer (HR, 0.74; 95%- CI, 0.71–0.77), liver cancer (HR, 0.73; 95%-CI, 0.55–0.98), gastric cardia cancer (HR, 0.78; 95%- CI, 0.64–0.95), kidney cancer (HR, 0.77; 95%- CI, 0.70–0.85), endometrial cancer (HR, 0.79; 95%- CI, 0.68–0.92), myeloma (HR, 0.83; 95%- CI, 0.72–0.95), myeloid leukemia (HR, 0.80; 95%- CI, 0.70–0.92), and bladder cancer (HR, 0.87; 95%- CI, 0.82–0.92). However, a higher level of leisure-time physical activity increased the risk of prostate cancer and malignant melanoma (HR, 1.27; 95%- CI, 1.16–1.40) [22].

Varied biological pathways have been found to illustrate the effect of physical activity levels on cancer risk. Pro-inflammatory cytokines, such as TNF-α, interleukin-1β (IL-1β), and IL-6, generated by adipocytes are elevated in adipose tissues. They could enhance the levels of serum amyloid A (SAA) and C-reactive protein (CRP) and, as a result, lead to tumorigenesis. Generally, regular physical activity is believed to have anti-inflammatory impacts by reducing pro-inflammatory cytokines and augmenting anti-inflammatory biomarkers. Moreover, elevated physical activity and reduced sedentary behavior can boost metabolic function by increasing insulin sensitivity and lowering fasting glucose. From another standpoint, the balance between reactive oxygen species and anti-oxidant defenses can be influenced by physical activity. Reactive oxygen species can result in DNA damage and mutations in tumor suppressor genes. Anti-oxidant defenses manufactured through repeated physical activity inhibit oxidative stress and restrain cancer cell development [23].

One explanation of the findings of our study is that since we applied self-reported questionnaires, recall bias and misclassification of physical activity level might interfere with results. On the other hand, based on the current evidence, the accurate lifetime mostly affecting the occurrence of oral cancers is not clear and not having a precise physical activity level at the critical time of occurrence of oral cancers, could be another reason for the controversy detected.

The limitations of this study included the questionnaire’s recall bias and small sample size. In the subsequent investigations, researchers are recommended to take advantage of objective physical activity appraisal methods, such as pedometer and accelerometer, to eliminate the recall bias. This study was conducted in limited population and future studies in other regions to verify the reliability and applicability of the research results, is suggested. In this study detailed smoking status and alcohol consumption was not obtained, comprehensive information regarding these issues is recommended in future studies. It is also recommended to conduct prospective cohort studies from childhood with long-term follow-ups and a higher sample size to discover the effect of physical activity on the risk of OSCC development.

## Conclusions

Based on the findings of this study, the average amounts of lifetime physical activity among cases and controls were similar. However, the overall time spent on physical activity regardless of its level and intensity can reduce the risk of OSCC. Considering the limited existing information, further evaluations and studies are mandatory to develop more precise conclusions.

## Supporting information

S1 Checklist (DOCX)

S1 Data (XLSX)
